# Corrosion inhibition properties of pyrazolylindolenine compounds on copper surface in acidic media

**DOI:** 10.1186/1752-153X-6-163

**Published:** 2012-12-31

**Authors:** Mehdi Ebadi, Wan Jeffrey Basirun, Hamid Khaledi, Hapipah Mohd Ali

**Affiliations:** 1 Department of Chemistry, Faculty of Science, University of Malaya, 50603, Kuala Lumpur, Malaysia; 2 Department of Chemistry, Faculty of Science, Islamic Azad University- Gorgan Branch, Gorgan, Iran; 3 Nanotechnology & catalysis research centre, Institute of postgraduate studies, University of Malaya, 50603, Kuala Lumpur, Malaysia

**Keywords:** Corrosion behaviour, Copper, Electro-corrosion, FESEM, Acid inhibition

## Abstract

**Background:**

The corrosion inhibition performance of pyrazolylindolenine compounds, namely 4-(3,3-dimethyl-3H-indol-2-yl)-pyrazole-1-carbothioamide (InPzTAm), 4-(3,3-dimethyl-3H-indol-2-yl)-1H-pyrazole-1-carbothiohydrazide (InPzTH) and 3,3-dimethyl-2-(1-phenyl-1H-pyrazol-4-yl)-3H-indole (InPzPh),) on copper in 1M HCl solution is investigated by electrochemical impedance spectroscopy (EIS), open circuit potential (OCP) and linear scan voltammetry (LSV) techniques.

**Results:**

The results show that the corrosion rate of copper is diminished by the compounds with the inhibition strength in the order of: InPzTAm> InPzTH > InPzPh. The corrosion inhibition efficiencies for the three inhibitors are 94.0, 91.4 and 79.3, for InPzTAm, InPzTH and InPzPh respectively with the same inhibitor concentration (2 mM).

**Conclusion:**

From the EIS, OCP and LSV results it was concluded that pyrazolylindolenine compounds with S-atom (with an amine group) have illustrated better corrosion inhibition performance compared to hydrazine and phenyl group.

## Background

Corrosion reactions are thermodynamically favourable on less noble metals and alloys when exposed to a corrosive environment such as chloridric acid. The inhibition of these reactions can be controlled by many types of organic and inorganic compounds [1-4], but organic compounds are the more common type of corrosion inhibitors. Most organic compounds which are efficient corrosion inhibitors contain functional groups which incorporate phosphorus, oxygen, nitrogen, sulfur atoms and multiple bonds [5,6]. The action of these inhibitors are closely related to factors such as: the types of functional groups, the number and type of adsorption sites, the charge distribution in the molecules and the type of interaction between the inhibitors and the metal surface [7]. A large number of organic compounds have been investigated as corrosion inhibitors for different types of metals [8-10]. With increased awareness towards environmental pollution and control, the search for less toxic and environment friendly corrosion inhibitors are becoming increasingly important.

**Figure 1:**
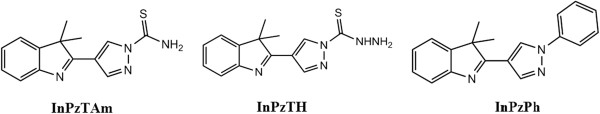
Molecular structure of the pyrazolylindolenine compounds.

We have recently reported the synthesis and the structures of new pyrazolylindolenine compounds which are 4-(3,3-dimethyl-3*H*-indol-2-yl)-pyrazole-1-carbothioamide (InPzTAm), 4-(3,3-dimethyl-3*H*-indol-2-yl)-1*H*-pyrazole-1-carbothiohydrazi (InPzTH) and 3, 3-dimethyl-2-(1-phenyl-1*H*-pyrazol-4-yl)-3*H*-indole (InPzPh) [11]. The corrosion inhibition properties of these compounds on copper surface in hydrochloric acid media are investigated using electrochemical impedance spectroscopy (EIS), open circuit potential (OCP), and field emission scanning electron microscopy (FESEM) techniques. The chemical structures of the compounds are shown in Figure 1. 

## Experimental

### Preparation of the pyrazolylindolenine compounds

The three pyrazolylindolenine compounds were synthesised through the reaction of 2-(diformylmethylidene)-3,3-dimethylindole with thiosemicarbazide (for InPzTAm), with thiocarbohydrazide (for InPzTH) and with phenylhydrazide (for InPzPh). The details of the synthetic and characterization methods are described elsewhere [11].

### Corrosion characterization techniques

All experiments were done using Cu plates of 99.9% purity (Goodfellow Cambridge) in 1 M HCl aqueous media containing various amount of pyrazolylindolenine compounds: InPzTAm (0–2.5 mM l^-1^), InPzTH (0–2.5 mM l^-1^) and InPzPh (0–4 mM l^-1^). The Cu plates (0.3× 1× 1 cm) were polished with emery paper (2000 grit), rinsed in distilled water and ultrasound in acetone to remove oily stains. A three-compartment cell was used in the corrosion measurements with a saturated calomel electrode (SCE) as the reference electrode and Cu plate and platinum wire with the same surface area as the working and counter electrodes respectively. Frequency response analysis (FRA) software was used in the EIS experimental and simulation process, while general purpose electrochemical software (GPES) was used in the linear scan voltammetry (LSV, Tafel) and open circuit potential (OCP) techniques. The software was installed in a computer interfaced with an Autolab (302 N) potentiostat/galvanostat instrument. The scan rate for LSV was 10 mV s^-1^, while the potential rage was between 0.3 V to −0.4 V). The EIS measurements were carried out at the OCP value with a frequency domain of 100 kHz-10 mHz with an amplitude of 5 mV. Prior to all analysis (EIS and LSV) the Cu plates were immersed in the corrosive solution (1 M HCl) containing different amount of inhibitors for 1 hr to obtain the OCP value. A JEOL JSM-840A field emission scanning electron microscopy (FESEM) instrument was used to capture the images of the copper surface after immersion in the corrosive solution for 1 week.

## Results and discussions

### Electrochemical impedance spectroscopy

**Figure 2:**
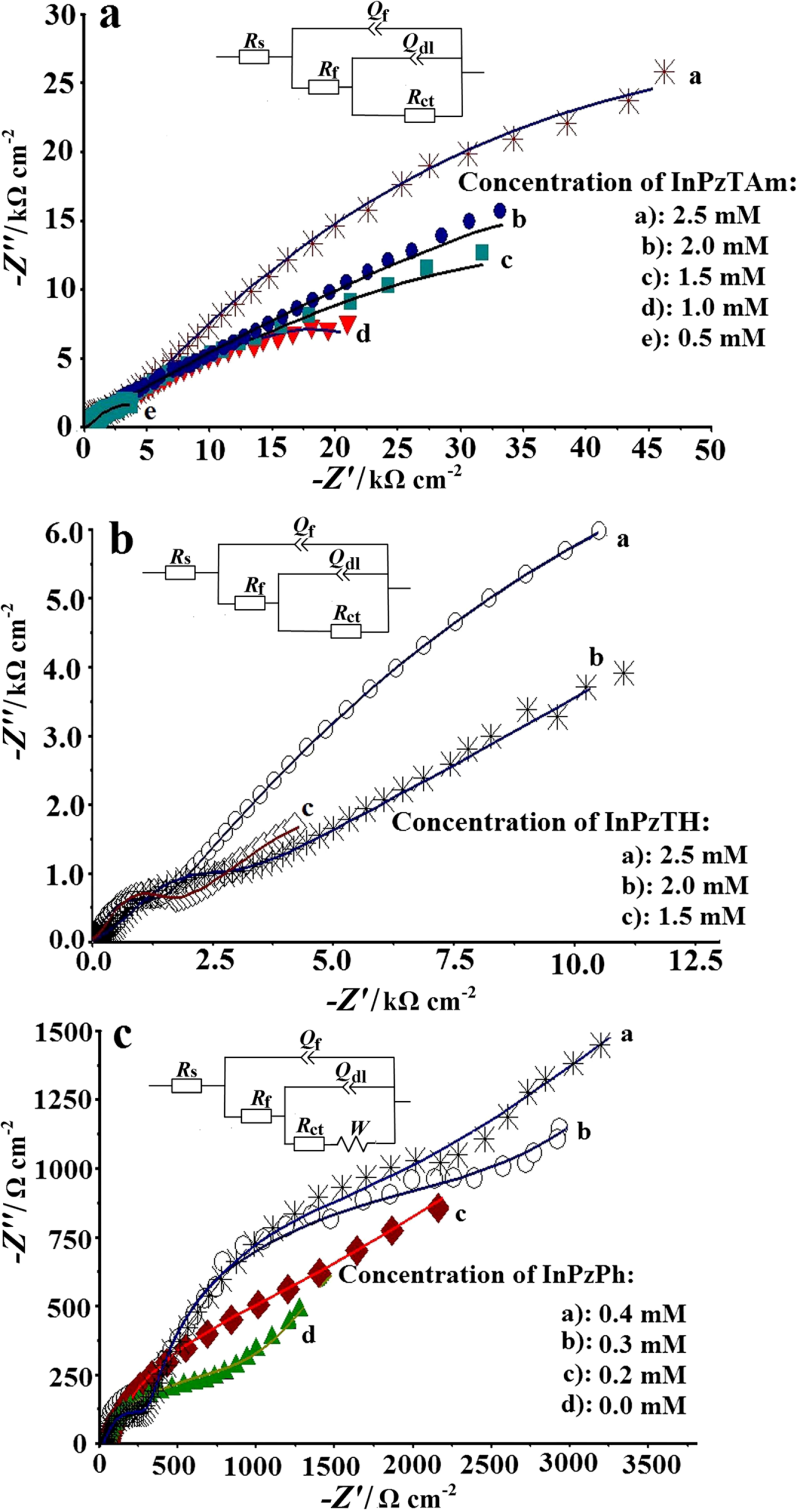
**The Nyquist plots (vs. SCE) for copper plates immersed in various amounts of pyrazolylindolenine compounds in 1 M HCl.** The symbols represent experimental data while the continuous line represent simulation.

The Nyquist and Bode plots are used to study the corrosion inhibition performance of the pyrazolylindolenine compounds. The Nyquist plots for the copper plates in corrosive media (1 M HCl) in the absence and presence of different amounts of the pyrazolylindolenine compounds are shown in Figure 2 (a, b and c). In the Nyquist plot, the diameter of the semicircles represents the charge transfer resistance (*R*_ct_) between the outer Helmholtz plane (OHP) and the electrode surface [12], and can be determined from the difference between the lower and higher frequency intercepts on the real axis *Z*_r_[13]. With the correct choice of the equivalent circuits, the closest fit to the experimental data can be done by simulation where the chi-squared value is minimised to 10^-4^ (Figure 2a-c). The solution resistance (*R*_s_) is the resistance between the working electrode (WE) and the counter (CE) and reference electrode (RE). The ability of the Helmholtz plane to accumulate charges can be defined as the double layer capacitance (*C*_dl_). The constant phase element (CPE) instead of *C*_dl_, is introduced in the simulation of the experimental data to obtain a more accurate fit. The impedance of the *CPE* (*Z*_CPE_) is a function of angular frequency (*ω=2πf*) and the magnitude of “*Q*” and “*n*”. Furthermore, *R*_f_ is the resistance of the inhibition film on the copper surface. Instead of the capacitance of inhibition film (*C*), a constant phase element (*CPE*, denoted as *Q*_f_ in the circuit) is introduced and it forms the first parallel combination. The impedance of CPE can be written as [14,15]:

(1) $$Z_{\mathrm{CPE}}=Q^{-1}{{\left(\mathrm{j\omega}\right)}^{-}}^{n}$$

The conversion of *Q* to *C*_dl_ is given by the following equation [16]:

(2) $$C_{\mathrm{dl}}=Q{\left(\omega_{\max}\right)}^{n-1}$$

where *ω*_max_ is the angular frequency when *Z* imaginary (*Z*_i_) is maximum and n is the phase shift and is the degree of surface in-homogeneity. The value of “*n*” is also related to the slope of the log ℜ *Z* ℜ vs. log *f* in the Bode plot, when *n*=1, then *Q = C*_dl_.

Figure 2 (a, b and c) shows that the semicircle diameter (therefore the charge transfer resistance, *R*_ct_) increases with the increase of the inhibitor concentration. The inhibition efficiency of the compounds are calculated and tabulated in Table 1.

(3) $$\eta \%=\frac{R_{\text{ct.(inhib.)}}-R_{\mathrm{ct}.\left(\mathrm{blank}\right)}}{R_{\mathrm{ct}.\left(\mathrm{inhib}\right)}}\times 100$$

where *R*_ct.(inhib.)_ and *R*_ct.(blank)_ are the charge transfer resistance in the presence and absence of the inhibitors respectively [17]. From Table 1 unlike the *R*_ct_, the change of the *C*_dl_ is not consistent with the increase of the inhibitor concentration, but is a function of the double layer thickness (*δ*). Therefore, the *C*_dl_ is affected by the changes in the double layer thickness (*δ*) [17], where *C*_dl_*=* (*εε*_0_*/δ*), where ε is the dielectric constant of the protective layer and *ε*_0_ the permeability of free space respectively. Khaled [18] has reported that the charge resistance of copper surface was increased from 0.58 (bare copper) to 11.77 kΩ/cm^2^ when N-(5,6-diphenyl-4,5-dihydro-[1,2,4] triazin-3-yl)-guanidine (NTG) (0.01 M) was added to H_2_SO_4_ (0.5 M) media. It was found that the synthesized compounds (specifically InPzTAm) has shown good corrosion inhibition efficiency on copper surface compared to previous results by other researchers [19-22].

**Table 1:** EIS simulation results of copper surface in 1 M HCl with the presence of different inhibitor concentration

**Elements→ Inhib.↓**	**Inhi. Conc. (mM l**^**-1**^**)**	***R***_**s **_**(Ω cm**^**2**^**)**	***R***_**ct **_**(kΩ cm**^**2**^**)**	***n***	***Q *mS.s**^**n**^**. cm**^**-2**^	***C***_**dl **_**(μF cm**^**-2**^**)**	***W *mS.s**^**1/2**^**. cm**^**-2**^
Blank	0.0	19.21	0.298	0.7142	7.35×10^-3^	149.3	4.05×10^-4^
InPzPh	1.0	357	2.044	0.7448	1.44×10^-4^	11.90	5.88×10^-4^
	2.0	27.2	2.562	0.8910	4.32×10^-5^	14.63	5.23×10^-3^
	3.0	59.2	2.923	0.9132	4.41×10^-5^	18.31	5.26×10^-3^
	4.0	28	4.32	0.9104	1.44×10^-4^	57.25	5.06×10^-3^
InPzTH	2.5	31.17	35.92	0.6573	1.55×10^-4^	2.27	
	2.0	36.2	21.14	0.6762	2.77×10^-4^	5.65	
	1.5	18.92	11.56	0.7060	4.05×10^-4^	15.49	
	1.0	27.23	1.8	0.852	4.83×10^-4^	17.31	
InPzTAm	2.0	30.40	174.5	0.8263	2.62×10^-6^	0.24	
	1.5	24.23	143.5	0.8782	1.18×10^-6^	0.23	
	1.0	17.39	68.7	0.8060	3.13×10^-6^	0.39	
	0.5	17.48	56.5	0.8089	3.03×10^-6^	0.27	

### Potentiodynamic measurements and thermodynamic calculations

**Figure 3:**
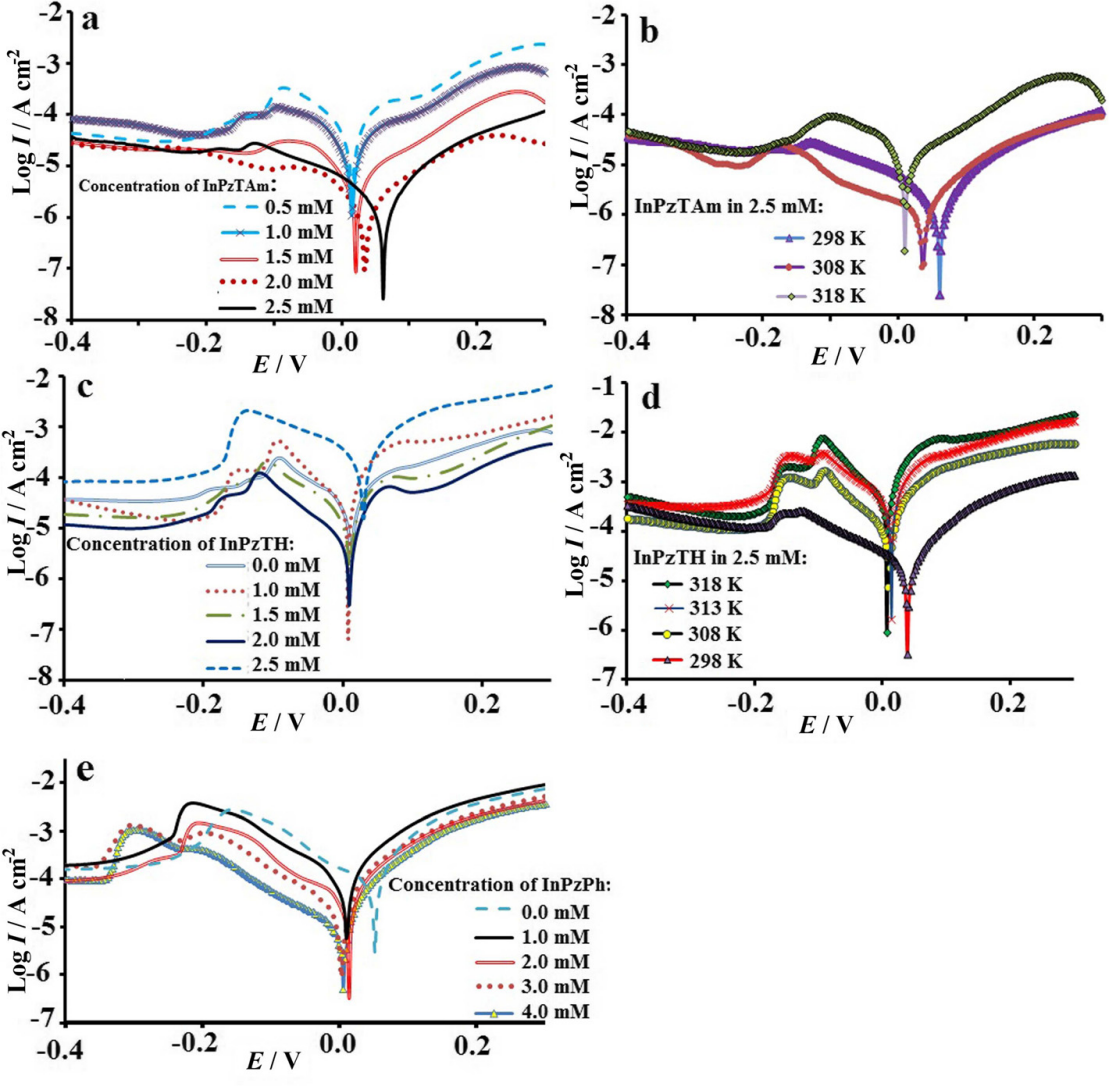
Tafel polarization curves (vs. SCE) for corrosion of Cu plates in 1 M HCl in the presence of different concentration of pyrazolylindolenine compounds (left side curves: a, c and e), Tafel plots in the right side (b and d) shows corrosion behaviour in the presence of 2.5 mM inhibitors at different temperatures.

Potentio-thermodynamic polarization is done on copper plates in 1.0 M HCl at room temperature containing various amounts of the inhibitor compounds (InPzTAm: 0.5-2.5 mM, InPzTH:1–2.5 mM and InPzPh: 1–4 mM), and is shown in Figure 3 (a, c and e). The electrochemical parameters such as corrosion rate (*CR*), corrosion potential (*E*_corr*.*_), corrosion current (*I*_corr._), Tafel slopes and the inhibition efficiency (*E%*) are obtained from the potentiodynamic measurements and given in Table 2. Li et al. [23] and Ferreira et al. [24] have suggested that a cathodic and an anodic type inhibitor will show *E*_corr._ shift of more than 85 mV with respect to the *E*_corr._ without the inhibitor, and a mixed type of inhibitor will show a shift of less than 85 mV. 

**Table 2:** Electrochemical impedance parameters for copper plates in 1 M HCl solution in the absence and presence of different concentrations of the pyrazolylindolenine compounds

	**Conc. Inhib. (mM)**	***E***_**corr. **_**(mV)**	***I***_**corr. **_**(A cm**^**-2**^**)**	***β***_**c **_**(mV/dec)**	***β***_**a **_**(mV/dec)**	***R***_**p **_**(Ω cm**^**-2**^**)**	***CR *(mm/y)**	***η%***	***K***_**ads. **_**(mol**^**-1**^**)**	***ΔG *(kJ mol**^**-1**^**)**	***θ***
Blank	0.0	0.6	19×10^-4^	184	144	368	4.694	-	-	-	-
InPzTAm	0.5	0.300	3.2×10^-4^	187	114	290	3.7	21.1	2.74×10^-4^	−6225	0.212
	1.0	0.013	3.7×10^-5^	132	125	190	0.43	90.8	9.9×10^-3^	−15633	0.909
	1.5	0.011	2.8×10^-5^	107	74	120	0.34	92.7	12.7×10^-3^	−32501	0.928
	2.0	0.008	2.4×10^-5^	64	72	81	0.28	94.0	15.7×10^-3^	−33552	0.940
	2.5	0.01	1.6×10^-5^	87	44	99	0.19	95.9	23.4×10^-3^	−44394	0.960
InPzTH	1.0	0.015	4.3×10^-5^	218	215	470	0.49	89.5	8.58×10^-3^	−15279	0.896
	1.5	0.02	5.8×10^-6^	95	138	970	0.67	85.7	6.01×10^-3^	−21595	0.857
	2.0	0.024	2.3×10^-6^	101	129	1420	0.40	91.4	10.73×10^-3^	−31666	0.915
	2.5	0.034	1.7×10^-6^	98	121	2900	0.21	95.3	20.33×10^-3^	−43542	0.953
InPzPh	1.0	0.015	7.0×10^-5^	193	89	100	3.12	33.9	0.51×10^-3^	−8235	0.340
	2.0	0.010	5.8×10^-5^	127	89	84	0.97	79.3	3.84×10^-3^	−26573	0.794
	3.0	0.002	3.3×10^-5^	115	64	97	0.78	83.3	5.02×10^-3^	−41852	0.834
	4.0	0.006	1.9×10^-5^	142	63	210	0.72	84.6	5.52×10^-3^	−56744	0.847

In this work the displacement range of *E*_corr._ for InPzTAm (Figure 3a) is 15.9-62.9 mV towards the anodic region, whereas InPzTH shows a 32.8-7.3 mV displacement to the anodic region (Figure 3c). Figure 3e shows that the Tafel curve has a small shift to the cathodic zone (< 85 mV) with the increase of InPzPh (54.4-5.2 mV). Therefore it can be concluded that the pyrazolylindolenine inhibitors (InPzTAm, InPzTH and InPzPh) show mixed type behaviour. From Figure 3a, the InPzTAm concentration of 2.5 mM shows maximum inhibition efficiency. The corrosion rate of the copper plate decreases from 4.69 mm y^-1^ to 1.9×10^-1^ mm y^-1^ with the addition of 0 to 2.5 mM l^-1^ for InPzTAm; from 4.69 mm y^-1^ to 2.1×10^-1^ mm y^-1^ with the addition of 0 to 2.5 mM l^-1^ for InPzTH; and from 4.69 mm y^-1^ to 7.2×10^-1^ mm y^-1^ with the addition of 0 to 4.0 mM l^-1^ for InPzPh. These results are consistent with the EIS results. The inhibition efficiency (η *%*) is calculated using the data from the corrosion potential (*E*_corr._), cathodic and anodic Tafel slopes (*β*_c_ and *β*_a,_ respectively) and corrosion current density (*I*_corr*.*_), and are tabulated in Table 2. From Table 2, larger differences in the cathodic slopes are shown by InPzTAm and InPzTH. This can be attributed to the thickening of the electrical double layer due to the adsorbed inhibitor molecules. According to Bockris and Srinivason [25], this behaviour is associated with the changes in the mechanism of the cathodic reaction, e.g. hydrogen evolution reaction (HER). On the other hand, there are no significant changes for the anodic and cathodic Tafel slopes (*β*_a_, *β*_c_, respectively) for InPzPh. It can be suggested that there are also no significant changes in the inhibition mechanism of cathodic and anodic reactions with the increase of the InPzPh concentration.

The change of the corrosion rate with temperature (298 to 318 K) is also examined on the copper plates. Figure 3 (b and d) shows that the activity of the inhibitors decreases with the increase of temperature from 298 to 318 K. This is due to the decrease of adsorption strength of the inhibitor on the metal surface with the rise in temperature. The adsorption of the inhibitor is dependent on many factors such as temperature, adsorption of solvent, type of ions, thickness of the electrical double layer and charge density. The surface coverage (*θ*) is an important factor which can be used to describe the type of adsorption mechanism of the inhibitors. The common isotherms which are used to calculate the surface coverage (*θ*) are [26]:

Frumkin isotherm: (*θ/*1*-θ*)*exp*(−2*Fθ*) *= K*_ads_. *C*

Freundlich isotherm: *θ = K*_ads_. *C*

Temkin isotherm: exp(*f.θ*) *= K*_ads_. *C*

**Figure 4:**
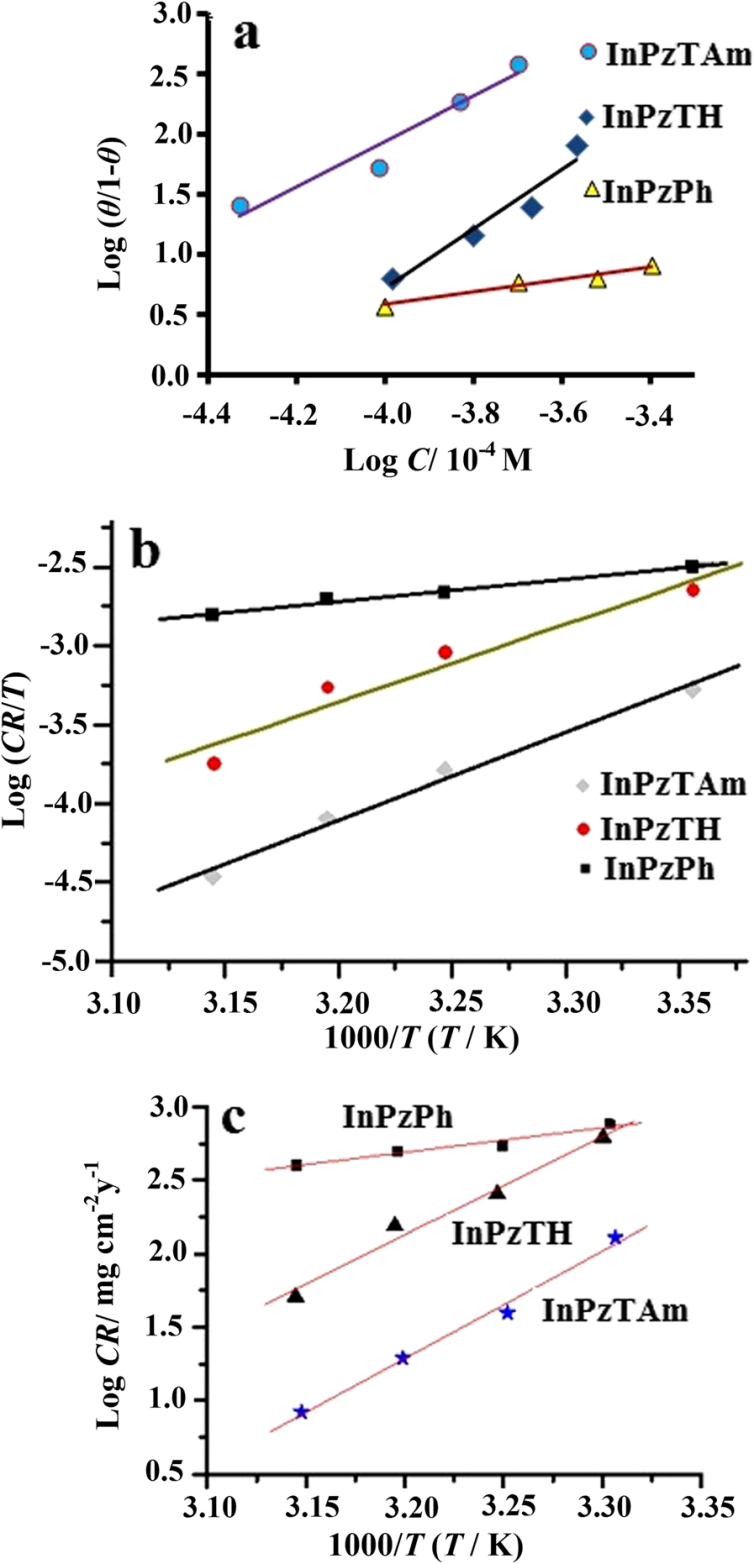
Adsorption isotherm plots in various amounts of pyrazolylindolenine compounds in 1 M HCl media a); log θ/θ-1 vs. log C, b) logCR/T vs. (1000/T), c); logCR vs. (1000/T).

Langmuir isotherm: (*θ/*1*-θ*) *= K*_ads_. *C*where *K*_ads._ is the equilibrium constant for the adsorption process, *C* is the inhibitor concentration, and *f* is the energic in-homogeneity. The Langmuir adsorption isotherm gives the best straight line for the inhibitors (Figure 4a: log*θ/*1*-θ* vs. log *C*). The calculation of *θ* value is given by [12]:

(4) $$\theta =\frac{C_{\mathrm{Ro}}-C_{R}}{C_{\mathrm{Ro}}-C_{\mathrm{Rm}}}$$

where *C*_Ro_, *C*_R_ and *C*_Rm_ are the corrosion rate of copper plates for the uninhibited, inhibited and the highest concentration of the inhibitor in 1.0 M HCl solution respectively. The straight lines in Figure 4a (log*θ/*1*-θ* vs. log *C*) suggest that the inhibitors at the metal/electrolyte interface follows the Langmuir adsorption isotherm. The thermodynamic parameters such as entropy of activation (*ΔS*), and enthalpy of activation (*ΔH*) and activation energy (*E*_a_) are calculated using the Arrhenius equation (Eq. 6) [13]:

(5) $$\log\left(\mathrm{CR}\right)=\frac{-E_{a}}{2.303\mathrm{RT}}+\lambda$$

(6) CR=RTNhexpΔSRexp−ΔHRT

where *T* is the temperature, *R* is the gas constant, *N* is the Avogadro number and *h* is the Plank constant. The diagram log(*CR/T*) vs. (1000/*T*) is plotted and *ΔH* and *ΔS* are calculated from the slope and intercept, from the values of (−*ΔH/2.303R*) and [log(*R/Nh*) + (*ΔS/2.303R*)] respectively (Figure 4b). It can be observed that the *ΔS* and *ΔH* are enhanced due to the increase of inhibitors in the corrosive electrolyte. The activation energy (*ΔE*) can be determined from the slope of log *CR* vs. (1000/*T*) in Figure 4c.

The thermodynamic parameters such as activation energy (*E*_a_) and the Arrhenius pre-exponential factor (*λ*) are obtained from the slope and intersection of the straight lines (Figure 4c). The linear regression coefficients (*R*^2^) are close to 1 for all three inhibitors. The values of *E*_a_ and *λ* for the inhibitor compounds are tabulated in Table 3, where the highest and lowest values are shown by the InPzTAm and InPzPh respectively. The higher value of *E*_*a*_ shows a lower corrosion rate this is also confirmed by the electrochemical tests in this work.

**Table 3:** Computed molecular parameters for the pyrazolylindolenine compounds

	**InPzTAm**	**SamplesInPzTH**	**InPzPh**
*∆S*_ads._ (kJ mol^-1^)	−16.25	−38.24	−137.76
*∆H*_ads._ (kJ mol^-1^)	−105.97	−94.62	−24.80
*λ* (mg cm^-2^ h^-1^)	−22.10 ±1.60	−19.24 ±2.19	−2.62 ±0.91
*E*_a_ (kJ mol^-1^)	7.34 ±0.49	6.68 ±0.68	1.67 ±0.28
*E*_HOMO_ (eV)	−0.2297	−0.2268	−0.2046
*E*_LUMO_ (eV)	0.19846	0.19864	0.18805
*∆E*= *E*_LUMO_ - *E*_HOMO_	0.03124	0.02816	0.01655

Gaussian software 9.0 is used to study the quantum chemical behaviour of the inhibitor. The quantum chemical properties of the inhibitors are studied by calculating the energy of the highest occupied molecular orbital (*E*_HOMO_) and the energy of lowest unoccupied molecular orbital (*E*_LUMO_). Prior to the calculation using the Gaussian 9.0, optimizations of the inhibitor molecules are done by the same software.

**Figure 5:**
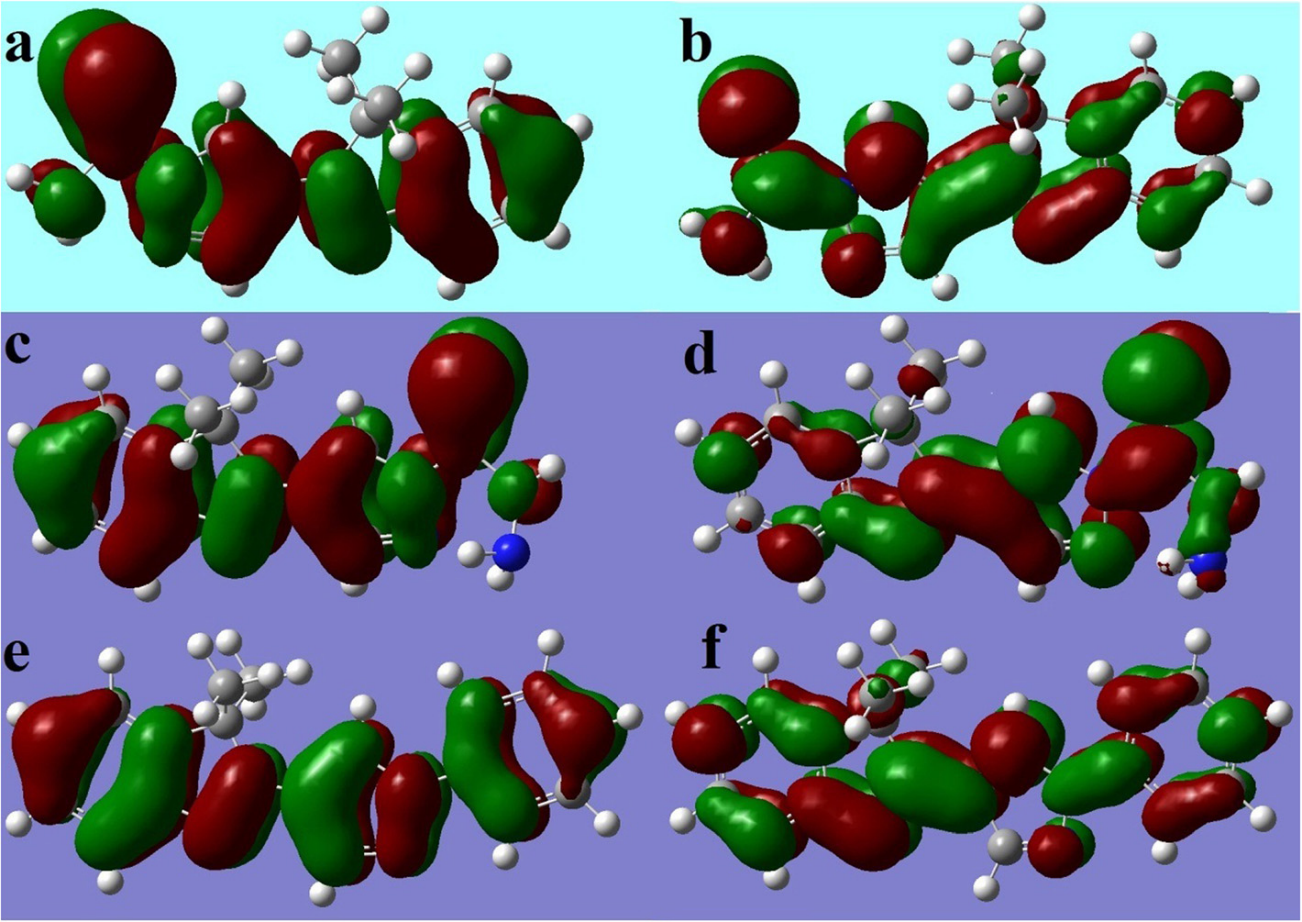
Frontier molecular orbital density distribution for InPzTAm (a; HOMO, b; LUMO), InPzTH (c; HOMO, d; LUMO) and InPzPh (e; HOMO, f; LUMO).

**Figure 6:**
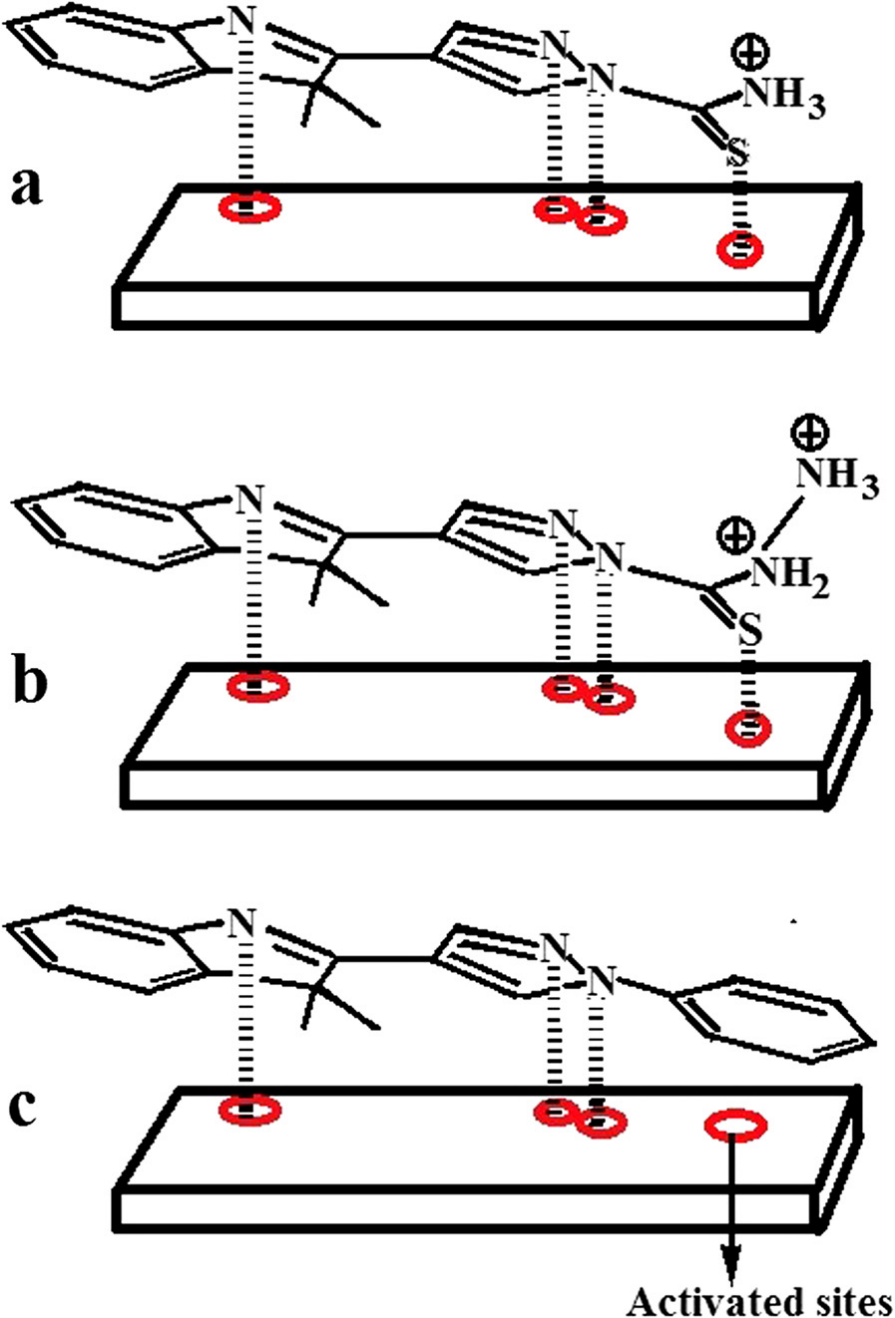
A schematic model for the adsorption of a): InPzTAm, b): InPzTH, c): InPzPh on copper surface.

**Scheme 1:**
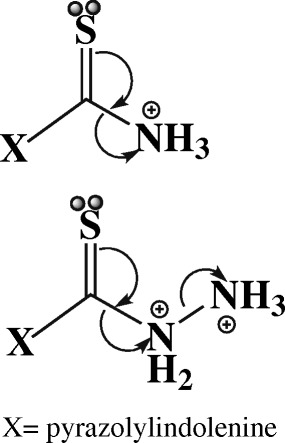
Structure and electron transfer mechanism of protonated InPzTAm and InPzTH, (X= pyrazolylindolenine).

The calculated *E*_HOMO_ and *E*_LUMO_ in Table 3 show that the *E*_LUMO_ and *E*_LUMO_ values are almost the same for InPzTAm and InPzTH. This is not unexpected since their molecular structures are almost similar. The *E*_LUMO_ values show that InPzTAm and InPzTH have good electron donor properties compared to InPzPh. This is due to the presence of sulfur atoms in both InPzTAm and InPzTH as shown in Figure 1, where the electron charge density is further away from the nucleus and can be donated easily for adsorption and bonding on the copper surface. However, the *E*_HOMO_ value for InPzPh shows good electron acceptor behaviour compared to InPzTAm and InPzTH. Frontier molecular orbital (FMO) density distributions for InPzTAm, InPzTH and InPzPh are shown in Figure 5. From the FMO results, the mechanism of inhibition on the copper surface can be described. It is a general assumption that the organic inhibitors are protonated in the acidic media. The adsorption of the inhibitors on the activated sites on the metal surface occurs by one or more of the following ways: (I) electrostatic interaction between the charged atoms of the inhibitors and the activated metal surface. (II) Unshared electron pair of the hetero-atoms (in hetero-cyclic molecules) and metal surface atoms with vacant d-orbitals. (III) Interaction of π-electrons with metal surface atoms. (IV) Interaction between protonated inhibitors with absorbed chloride ions. In our previous work [27], we have suggested that the nitrogen protonated caffeine molecule adsorbed the chloride ion while the electron pair of N-atom was bonded with the metal surface, thus preventing the chloride ion from attacking the metal surface. The adsorption mechanisms of the synthesized pyrazolylindolenine compounds on the copper surface are shown in Figure 6. The different strengths in the inhibition efficiency of these compounds are due to the different functional groups which are bonded with Pyrazolylindolenine base. The InPzTAm and InPzTH compounds have similar chemical structure, but InPzTAm shows better inhibition efficiency. Scheme 1 a shows that the inter-electron transfer from S-atom to the amine protonated group which decreases the electron density in the S-atom. Compared to scheme 1 a, the Scheme 1 b shows that both N-atoms (in Hydrazine group) are protonated hence more electrons from the S-atom can be transferred. The S-atom in InPzTAm with higher electron density thus better electron donor capabilities shows better corrosion inhibition properties compared to InPzTH with less electron density. The InPzPh shows the weakest corrosion inhibition efficiency which is due to the absence of the C=S group. 

The hydrazine group in InPzTH with two N-atoms and the amine group in InPzTAm with one N-atom have slight difference in energies. Figure 5a and c show higher electron density in the vicinity of the N-atom in InPzTAm compared to N-atoms in the hydrazine group in InPzTH. Thus InPzTAm can be absorbed on copper activated sites via the donation of the unshared electron pair from the N and S-atoms to the copper metal. It was found that more electron density transfer from inhibitors to metal surface results in increased adsorption of the inhibitor molecules on the surface, thus better corrosion protection properties. Table 3 shows that the Δ*E* for compounds is in the order of InPzTAm > InPzTH > InPzPh. It can be shown that the electron transfer process in InPzTAm is faster compared to the other compounds.

### OCP measurements

**Figure 7:**
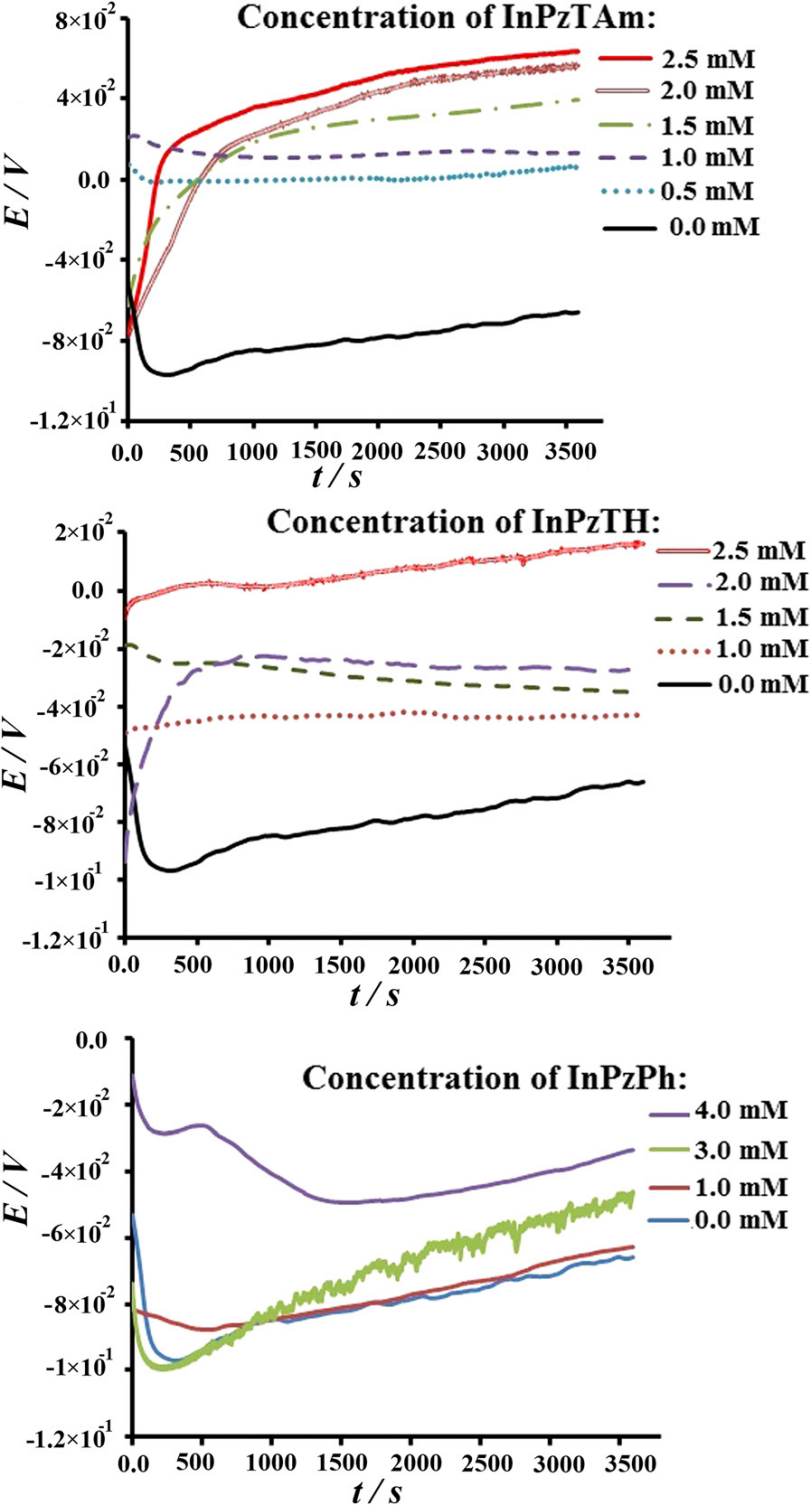
OCP measurements (vs. SCE) with time for copper plates with different concentration of the inhibitor compounds in 1 M HCl media for 1 h.

The changes of the open circuit potential (OCP) with time for the copper electrode in 1 M HCl solution in the absence and presence of various amount of inhibitor (InPzTAm, InPzTH and InPzPh) concentration at room temperature are shown in Figure 7 (a, b and c). Generally, the OCP variation with time with different amount of inhibitor shows a similar behaviour. Upon the immersion of the electrode in the solution, an accentuated displacement of OCP towards negative values was observed. The quick changes in the OCP curves could be due to the initial dissolution process of the oxide film formed on the bare copper surface. Soon afterwards (approximately 500 sec), the OCP increased towards positive regions while the metal surface was passivated due to the adsorption of the inhibitor compounds on the activated sites of Cu surface. It can be observed that the OCP shifts to more noble potentials with the increase of the inhibitor concentration. With the same concentration for all three inhibitors, the shift of the OCP towards more noble potentials is in the order of InPzTAm > InPzTH > InPzPh. The electrolyte has a high concentration of Cl^-^ and the Cl^-^ has a strong tendency to adsorb on the cathode surface, thus the local corrosion could be due to changes in the surface polarization [27]. With the increase of the inhibitor concentration in the corrosive electrolyte, the aggressive behaviour of Cl^-^ is quenched due to the increased adsorption of the inhibitors on the copper surface, thus the OCP moves towards noble potentials. Surface coverage (*θ*) is calculated and tabulated in Table 2. It shows that the increase of surface coverage is in the order of: InPzTAm > InPzTH > InPzPh, thus the adsorption of the inhibitors are also in that respective order. 

From Figure 7, the lowest shift towards positive potential is for InPzPh due to the low surface coverage while the largest shift of towards positive potential is for InPzTAm. This phenomenon can be ascribed to the decrease of the charge transfer and subsequently mass transport of the Cl^-^ decreases with the formation of the inhibitor adsorption on the copper surface. From the measurements of surface coverage *θ* and *K*_ads._ the strength of the inhibitor adsorption on the copper surface is in the order of InPzTAm > InPzTH >InPzPh.

### FESEM analysis

**Figure 8:**
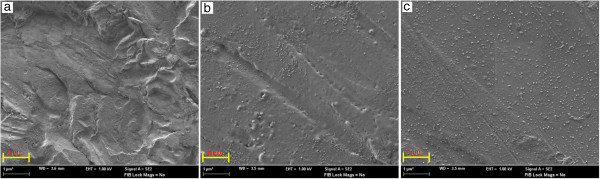
FESEM images (10000X) of the copper surface after immersion in 1.0 M HCl solution for 10 days with: a) the absence of inhibitors, b) 2.5 mM InPzTH and c) 2.5 mM InPzTAm.

FESEM images of the copper surface are taken to establish a link between the surface morphology and the experimental results obtained for the inhibition performance of the InPzTAm and InPzTH compounds. Figure 8 (a, b and c) are surface images of copper plates which were immersed in 1.0 M HCl, 1.0 M HCl + 2.5 mM InPzTH and 1.0 M HCl + 2.5 mM InPzTAm for 10 days, respectively. Figure 8a shows the presence of cavities and depth roughness on the copper surface after 10 days of immersion time in 1.0 M HCl, where the surface is strongly damaged in the absence of the inhibitors. On the other hand, surface images of 8b and 8c show that cavity formation and surface roughness on the copper surface has decreased with the presence of InPzTAm and InPzTH in the corrosive media (1.0 M HCl). The Cu surface with the presence of InPzTH (2.5 mM) shows few pit formation after immersion in 1.0 M HCl for 10 days (Figure 8b). The Cu surface with the presence of the InPzTAm did not show pit and cavity formation after 10 days of immersion time in the corrosive solution. The FESEM images for both inhibitor compounds conforms to the experimental results obtained in the previous sections.

## Conclusion

The EIS, OCP and LSV measurements show that all three pyrazolylindolenine compounds (InPzTAm, InPzTH and InPzPh) give good inhibition performance against copper corrosion in 1 M HCl solution. OCP measurements show that the OCP moves towards more noble potentials with the increase in the inhibitor concentration. Tafel polarization experiments show that the *R*_p_ also increases with the increase of inhibitors in acidic media. EIS simulation of the experimental data shows that InPzTAm and InPzTH have the same equivalent circuit but the Warburg element is present in the equivalent circuit for the InPzPh compound. The corrosion inhibition performance of these compounds for the same concentration (2 mM) is *η* = 94.0%, *η* = 91.4% and *η* = 79.3% for InPzTAm, InPzTH and InPzPh respectively. From the EIS, OCP and LSV results, the higher corrosion efficiency for both InPzTAm and InPzTH compared to InPzPh is due to the presence of sulfur atoms in those compounds. From quantum chemical calculations, the presence of the sulfur atoms promote better electron donor ability for both compounds which give higher inhibition efficiency.

## Abbreviations

InPzTAm: 4-(3,3-dimethyl-3H-indol-2-yl)-pyrazole-1-carbothioamide; InPzTH: 4-(3,3-dimethyl-3H-indol-2-yl)-1H-pyrazole-1-carbothiohydrazide; InPzPh: 3,3-dimethyl-2-(1-phenyl-1H-pyrazol-4-yl)-3H-indole; EIS: Electrochemical impedance spectroscopy; OCP: LSV: Open circuit potential, linear scan voltammetry; FESEM: Field emission scanning electron microscopy; SCE: Saturated calomel electrode; FRA: Frequency response analysis; GPES: General purpose electrochemical software; OHP: Outer Helmholtz plane; WE: Working electrode; CE: Counter electrode; CPE: Constant phase element; HER: Hydrogen evolution reaction; *E*_HOMO_: Occupied molecular orbital; *E*_LUMO_: Unoccupied molecular orbital.

## Competing interests

The authors declare that they have no competing interests.

## Authors’ contributions

ME completed the laboratory work, data treatment and drafted the manuscript. HK and HMA synthesized the inhibitors. WJB coordinated the study, data analysis and edited the text. All authors have read and approved the final manuscript.
